# Supplementary figures and images for: miRNA-mediated loss of m6A increases nascent translation in glioblastoma
Source: PLoS Genet. 2021 Mar 8;17(3):e1009086. doi: 10.1371/journal.pgen.1009086 (PMC7971852; doi:10.1371/journal.pgen.1009086)

**Supporting Information**

**Fig A**


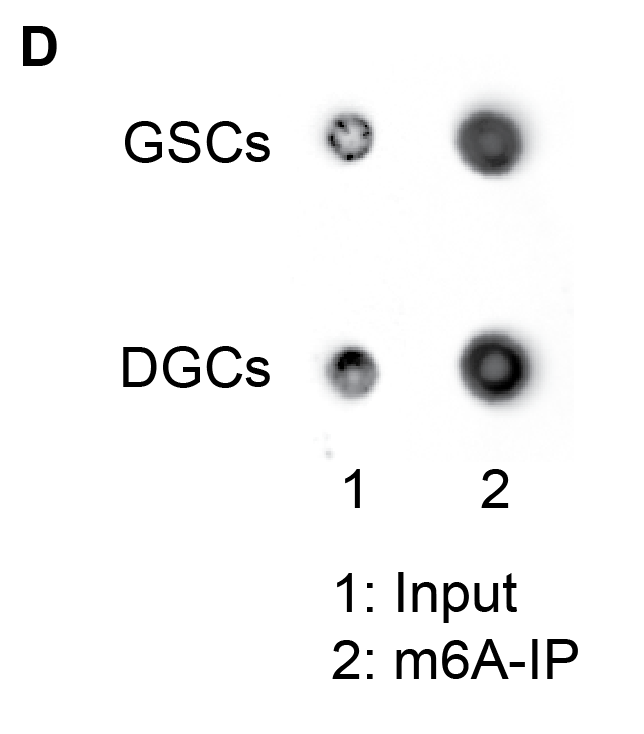


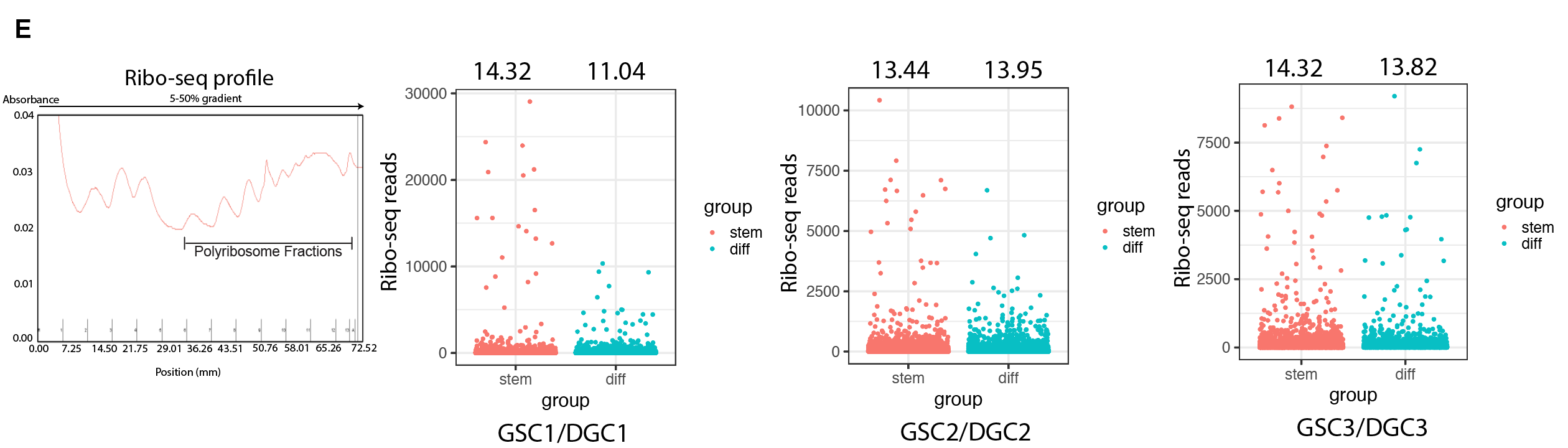


**Fig B**


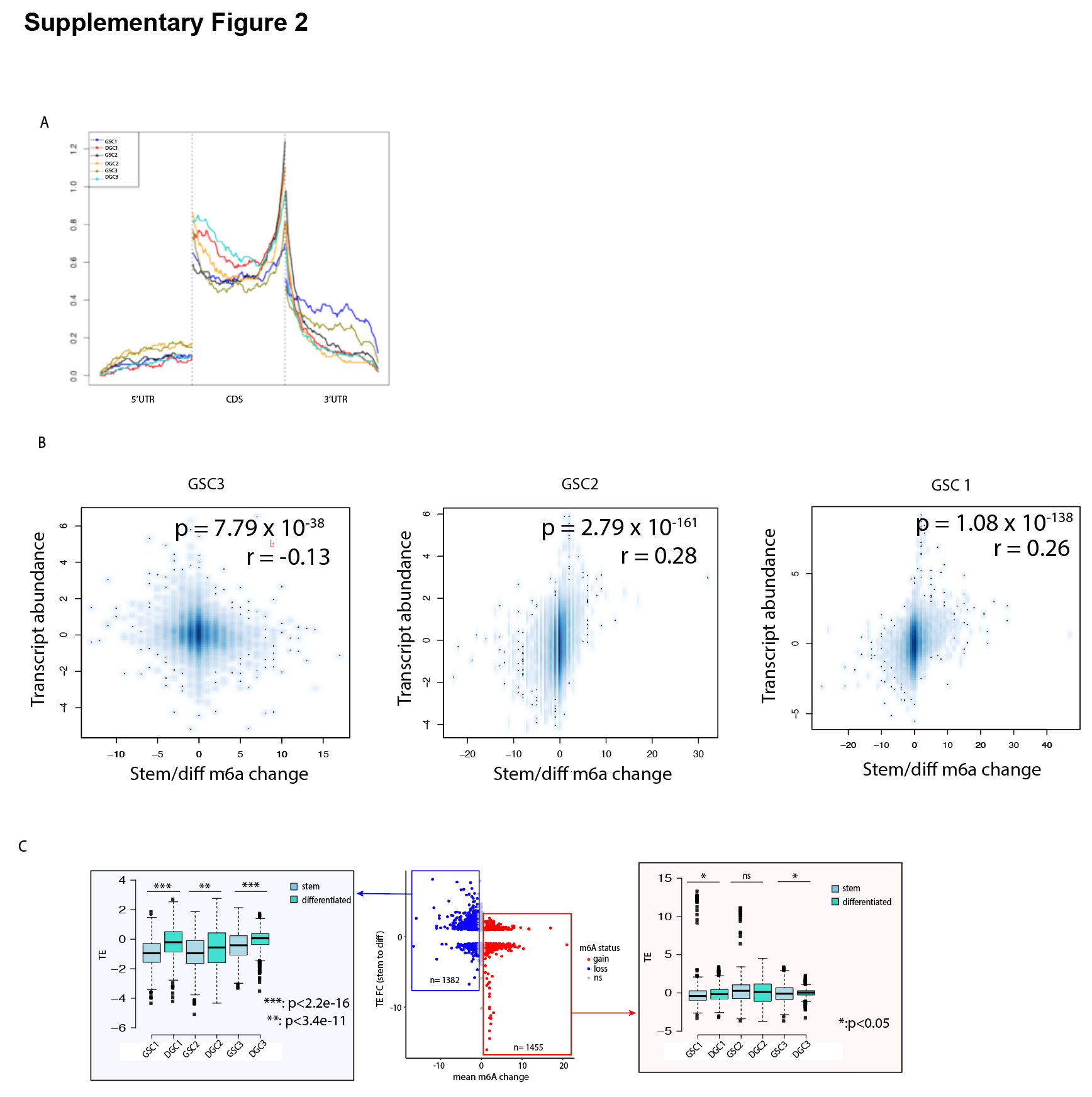


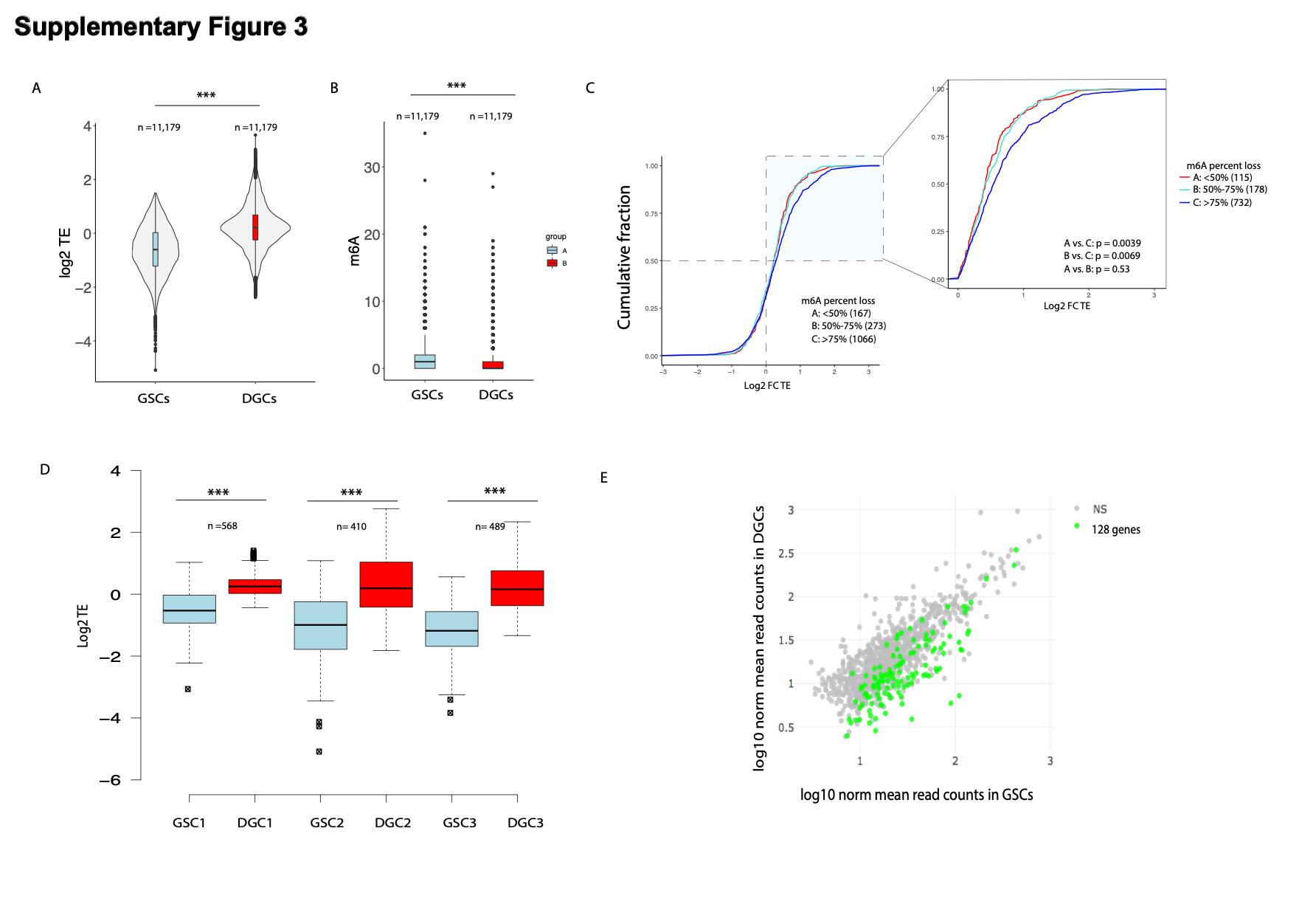


**Fig C**

**Fig D**


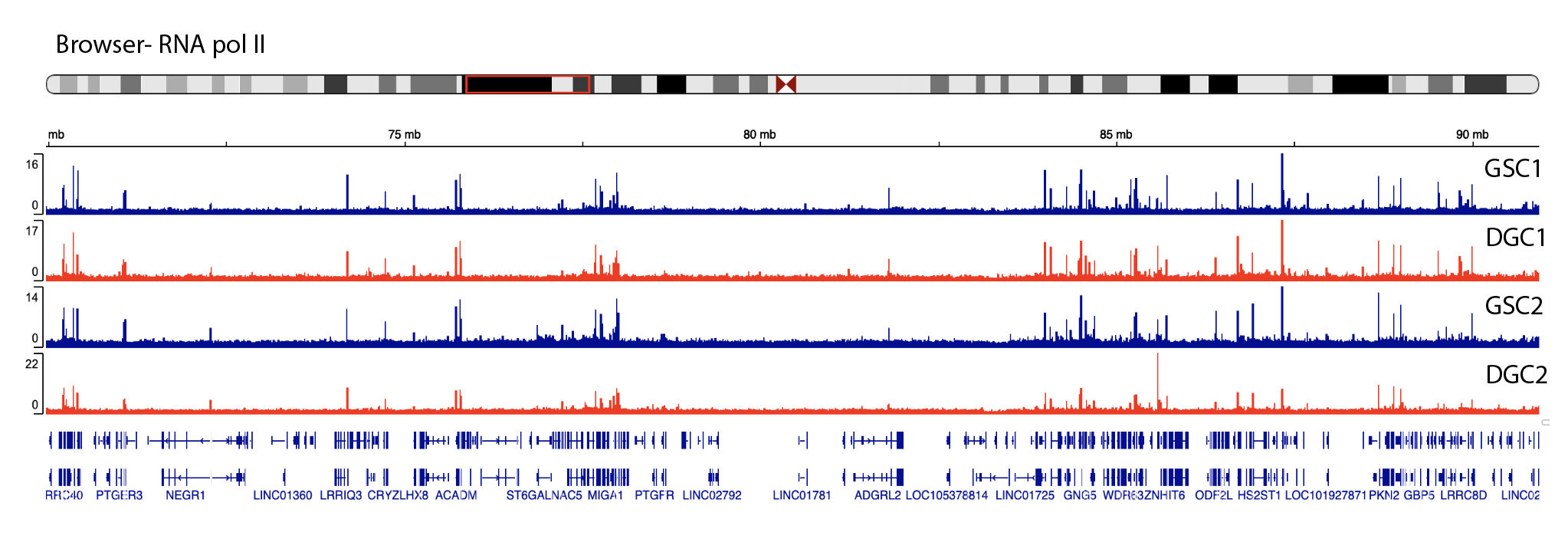


C

**
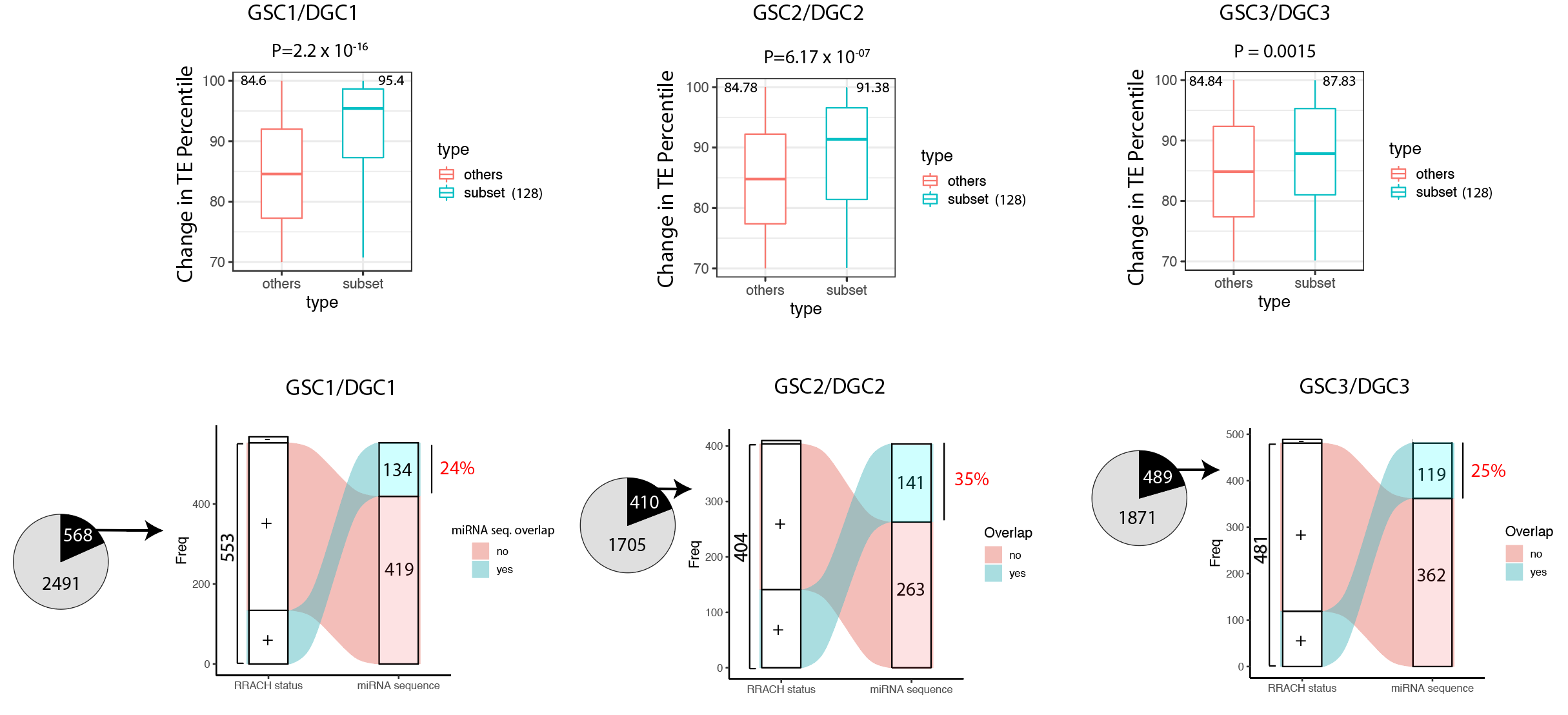
**

D

E


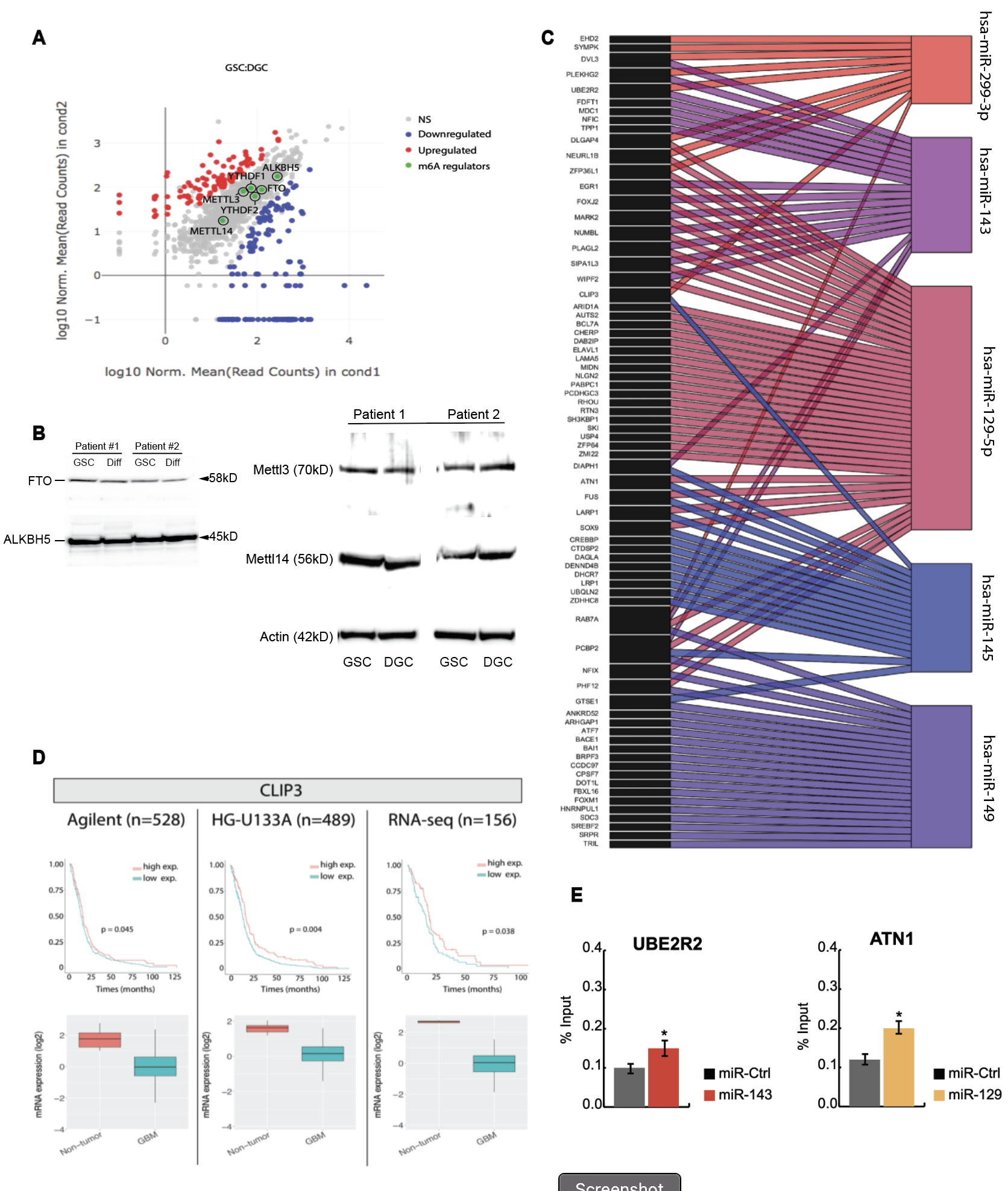
**Fig E**

**Fig F**


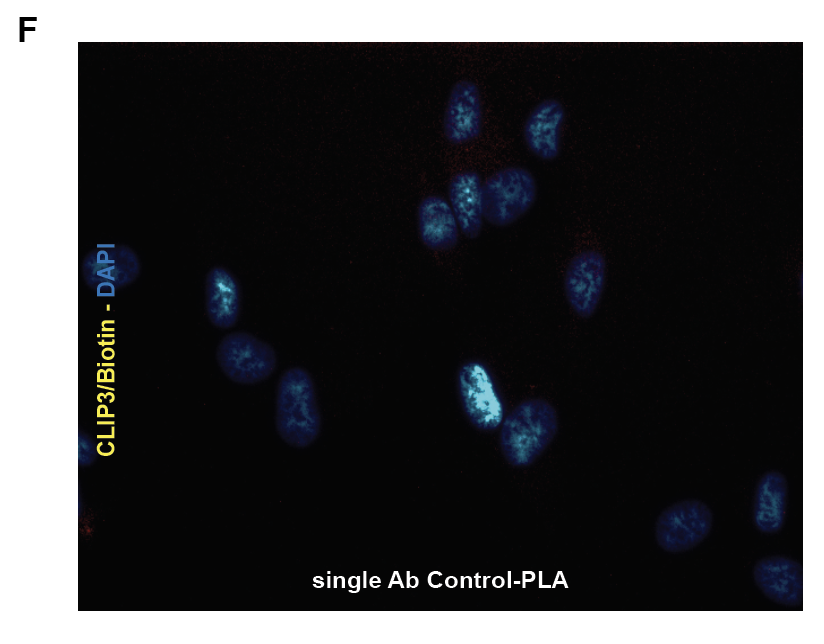

Supplement: S1 Text — Supporting figures A-F. Fig A: Characterization of CSCs and differentiated cells. A) CSCs express stem cell specific transcripts (CD133, Sox2, Olig2), which they completely lose (CD133, Olig2) or downregulate (Sox2) after differentiation for 7 days by removal of EGF, bFGF and Heparin and addition of 10% serum. Moreover, following differentiation the glioma cells gain expression of GFAP, which was not expressed in CSCs. The graph presents representative RNA-seq data from one CSC line. The same analysis has been performed for all CSCs and differentiated glioma cells. B) Limiting dilution assay to determine the self-renewal ability of GSCs. The experiments were repeated six times and significance was calculated with a Chi-square test (p<0.008). C) Orthotopic xenograft transplantation of CSCs in nude mice results in formation of invading glioblastomas, verifying the tumor-forming ability of the CSC lines. Image shows a HuNu positive glioblastoma 4 weeks after the transplantation of 150,000 CSCs. Hematoxylin was used as counterstain. Xenograft transplantations to examine tumor forming ability of CSCs are routinely performed for each newly isolated CSC line. D) Representative m6A dot blot following m6A RIP in GSCs and differentiated cells shows enrichment of m6A compared to input. E) Representative Ribo-seq profile of GSC. Ribo-seq reads distribution and median between stem and differentiated progenies showing that median values and read distribution are reproducible across experiments. Fig B: Methylation and Transcriptome Profile. A) Distribution of genome wide m6A peaks in GSCs and differentiated progeny divided in 5’UTR, CDS and 3’UTR peak regions. B) C) D) Mean m6A change vs. TE FC scatterplot and transcripts dichotomized into m6A loss (n = 1382) and gain (n = 1455) groups based on mean m6A change during differentiation. Transcripts TE distributions shown by mean m6A loss and gain in individual GSCs and corresponding DGCs. Fig C: A) Log2 TE comparison of GSCs and DGCs [file pgen.1009086.s001.docx]
