# Supplementary material for: miRNA-mediated loss of m6A increases nascent translation in glioblastoma
Source: PLoS Genet. 2021 Mar 8;17(3):e1009086. doi: 10.1371/journal.pgen.1009086 (PMC7971852; doi:10.1371/journal.pgen.1009086)
Supplement: Attachment — Submitted filename: Responses to reviewers comments-PLOS GENETICS.pdf [file pgen.1009086.s003.pdf]

Responses to reviewer comments (PGENETICS-D-20-01310)

Dear Drs. Ruzov and Bickmore,

On behalf of all co-authors, I am sending you our responses to reviewer comments of our manuscript (PGENETICS-D-20-01310) entitled: "**miRNA-mediated loss of m^{6}A increases nascent translation in glioblastoma**".
We would like to thank the reviewers for their constructive criticism of our work. In response to their comments, we have performed new experiments, edited the relevant figures of the paper and added new data in the main body of the manuscript and in supplementary section. We strongly believe that the revised manuscript answers all the reviewer's questions and should be now suitable for publication in PLOS GENETICS.

In detail, our point-by-point response:

Reviewer 1:

1- At the heart of the manuscript is the MeRIP-Seq experiment conducted to identify m6A methylated transcripts. Multiple figures do not contain any validation of the MeRIP-Seq approach. In particular, can the authors show that they are effectively pulling down m6A methylated transcripts using ideally mass spectrometry to show enrichment of m6A in pull-down vs input OR at least a dot blot approach to indicate to some level their IP is working. Can the authors document if the efficiency of m6A IP compatible between experiments in different figures (figure In Supp. Fig 2C, how does the input look like?
We provide a representative dot blot from MeRIP on GSCs and differentiated cells showing enrichment of m6A relative to input following m6A IP. This was performed in samples used for both Figure 1 and Supp. Fig 2C. The dot blot has been added to supplementary Figure 1D and explained on Page 6 of the revised manuscript.

2- Similarly, can the authors show that their ribosome profiling experiment is working as expected by showing actually the profile between experiments. As it stands the manuscript mentions both MeRIP-Seq and Ribosome profiling experiments were conducted without actually showing any validation that these methods work in the hands of the authors to a degree that is comparable with other studies.
We have added Ribo-seq profile of GSC and differentiated progenies. A sample ribo-seq profile is depicted on Supplementary Figure 1E and page 6 of the revised manuscript. We also show ribo-seq reads distribution and median between stem and differentiated progenies showing that median values and read distribution are reproducible across experiments.

3- What is the relation between m6A change and transcript abundance between GSCs and DGCs?
Change in m6a levels was quantified per transcripts per sample by calculating the difference in total m6a per transcript between both differentiated and stem cells. Transcript abundance was quantified by obtaining the fold change between both cell states. Pearson correlation was

subsequently conducted on all GSCs. Key findings: Shows that correlation between transcript abundance and m6a levels may be GSC dependent as GSC1 shows a trend opposite of GSC2 and GSC3. GSC1 exhibit a negative correlation between m6a and transcript abundance, while GSC2 and GSC3 show the opposite. These data and analysis have been added to the revised paper page 7 and Supplementary Figure 2B.

4- "Although median increase in TE was noted with both m6A loss and gain, there is a relatively more substantial increase in median TE in all DGCs in the subset of transcripts with mean m6A loss. In comparison, the transcripts with mean increase in m6A show a blunted non-generalized, increase in median TE (Supplementary Figure 2D). Taken together, these findings suggest a stronger link between m6A loss and TE increase during GSC differentiation."
From the above statement it is not clear (1) if there is a statistically significant difference in TE among genes that either loose or gain at least one m6A peak and (2) what authors mean by "blunted non-generalised increase" in contrast to "relatively substantial increase". I would expect to see some statistical tests to test hypothesis here and more clear language.
We re-worded the description of the analysis and results and provide all details regarding the statistical methods used and the p values as the reviewer suggested. Revised manuscript page 8 and supplementary figure 2C.

5- In supplemental figure 3, authors show that for the top 40% genes, there is a reduction in m6A and increase in TE between GSCs and DGCs. This is rather confusing, are the authors taking top 40% genes that show TE increase and plot that they indeed have TE increase? What about the rest of the genes? What is the profile for m6A status?
Figure 2B depicts a trend of decrease in median m6a with increasing TE that is notable at the 60th percentile change in TE ranking. 11,179 total transcripts across patients fall within the top 40 percent of transcripts which have an increase in TE and gradual median m6a loss. Figure 2B needs statistical support to support that these top 40th change in TE ranking percentile do indeed as a whole experience a statistically significant increase in TE and loss of m6a. The Supplemental figure 3A and 3B provide such support.

6- If a gene is showing increased translation efficiency, how likely it is that the said gene loses its m6A more than 75% as opposed to showing no change or increase in m6A?
Figure 2C and supplemental 3C collectively address this question. Figure 2C shows the distribution of loss, gain, no change in m6a in the top 30 percent transcripts with increase in TE ranking. It also shows that generally the majority of transcripts do not undergo change in m6a status. However, a substantial fraction of transcripts loses m6a peaks. Supplemental figure 3C assesses the relationship between percent m6a peak loss and TE. It shows that the majority of transcripts that lose m6a indeed lose more than 75% of their peaks during differentiation. Furthermore, when assessing transcripts with increase in TE, statically significant increase in TE occurs when samples experience 75% loss or greater. Of the transcripts that show increase in TE the majority lose 75% or more peak on average.
Of note- supplemental figure 3C is based on average change in TE and m6a, ribodiff package allowed for group-based comparison between GSCs and DGCs. The data therefore suggests

that, on average, the majority of transcripts that experience significant increase in TE following differentiation exhibit significant m6a loss greater than 75% m6a loss.

7- "In addition to the 70th percentile cut-off, we imposed a methylation cut-off of 2 peak loss in order to capture all transcripts significantly fitting the m6A loss and increase in TE trend." Previously authors used a 60th percentile and at least 1 m6A loss as a criteria. Why the need for change? This is not sufficiently explained.

This part of the manuscript discusses the organic progression of the analysis. The trend in median m6a loss initially occurs at the 60th percentile change in TE ranking. We decided to adopt more stringent parameters in our algorithm in order to unmask the mechanism presented in this study. For instance, instead of 1 peak loss for m6a loss, we chose 2 and instead of selecting the exact point of inflection at which median m6a depreciates, we selected the next percentile set which corresponded to the 70th percentile or transcripts with top 30 percent increase in TE.

8- Throughout the manuscript authors suggest that the m6A is lost on transcripts. It is important the authors clarify if they mean these transcripts are demetyhlated or they are not methylated when new transcripts are made. I suppose this cannot be distinguished with current data.

With the current data and analysis, we cannot claim that these transcripts are not methylated when made. We clarified this in the revised manuscript page 10.

9- No data is presented to show that RNA pol II ChIP experiments are working effectively across samples. Can the authors at least provide example regions with input and RNA pol II read peaks?

RNA Pol II ChIP-seq peaks are presented on supplementary figure 4 of the revised manuscript as the reviewer suggested.

10- In Figure 3, authors should also analyse all m6a regions and compare to 128 transcripts that follow a desired trend. This would provide evidence if these transcripts are unique and regulated differently or similar miRNA binding sites can be found in all m6A transcripts which would indicate additional mechanisms for the 128 transcripts.

We calculated the change in TE percentile between the 128 common transcripts and other transcripts with top 30% increased TE percentile. The top transcripts with the top 30% increased TE during differentiation were collected and grouped into 128 common transcripts and others (transcripts in the top 30% but that did not follow the m6a/TE trend across all GSCs). Wilcoxon test was performed on the change in TE percentile of the 128 common transcripts versus other top 30% non-common transcripts across all GSCs. Key finding: The 128 common transcripts experience the greatest increase in TE amongst the top 30% most efficiently translated transcripts. These data are presented in supplementary figure 4D and page 11 of the revised manuscript as the reviewer suggested.

Next, we determined the fraction of transcripts with m6a loss and increase in TE whose miRNA binding sequence overlaps a RRACH motif. All transcripts with m6a loss were collected per

patient. A group of transcripts with the top 30% TE percentile increase that have undergone significant peak loss (equal to or greater than 2 peak loss) were obtained (GSC1: 568 /3059; GSC2: 410/2115; GSC3: 489/2360). Of these transcripts, the majority, between 97% to 98%, was found to have a RRACH motif sequence and from those with a RRACH motif, between 24% and 35% had a RRACH motif overlapping a miRNA binding sequence. (GSC1: 134/553; GSC2: 141/404; GSC3: 119/481). Key findings: 24% to 35% of the transcripts that experience m6a loss and increase in TE during GSCs to DGCs transition have miRNA binding sequence overlapping the RRACH motif. This analysis and data were added to the revised manuscript Supplemental Figure 4E.

11- Figure 4A should include anti-FTO western blot to show that FTO is expressed, upon IP it is depleted from input and enriched in IP fraction to show that the IP is working effectively. In addition, is the FTO - AGO1 interaction RNA dependent? To test this authors should do the IP in the presence and absence of RNAse digestion.
We have repeated all IPs and included FTO WB as the reviewer suggested. Results are included in revised Figure 4A of the revised manuscript.
We have done the FTO IP in the presence and absence of RNase followed by WB for FTO and Ago1 to show if the complex formation is RNA dependent as the reviewer suggested. The results are included in Figure 4B and page 13 of the revised paper.

12- For Figures 4D, and Supp. Fig 5E what is the proof that authors are not immunoprecipitating more FTO after miRNA over expression?
We have added FTO WB in Figure 4E of the revised manuscript showing equal expression of FTO in miR-Ctrl and miR-145 RIPs as the reviewer suggested.

13- In Figure 5B and E, authors should present the single antibody only controls to clearly present the background level signal.
Antibody only control is included in Supplementary Figure 6F of the revised manuscript as the reviewer suggested.

14- Figure 5E, can the authors show that the mir-145 antagomir effect is specific to CLIP3 translation and not generally affecting all translation in the cells?
We don't think that this will add valuable information to the paper. Inhibition of miR-145 will affect translation of other miR-145 target transcripts through inhibition of the classical miR-based mRNA degradation pathway. However, the effect on CLIP3 does not depend on miRNA mediated mRNA degradation or stability but rather on the proposed m6A mediated regulation of translation as shown explicitly in the paper. Accordingly, we believe the focus should only be on transcripts that are subject to this m6A-mediated regulatory mechanism.

15- Overall, the methods section lacks sufficient detail in some parts;
- m6A RNA IP section; how is it possible to repeat these experiments with the information given here? How much antibody? What were the controls? How much RNA?
- where is the method for polysome fractionations?

- western blots; what are the antibody concentrations? How much protein loaded?
- etc.

We have added sufficient details in the Methods section as the reviewer requested.

Reviewer 2:

1. The authors should consider providing western blot evaluation of m6A demethylases (ALKBH5/FTO) and see whether their conclusion that loss of m6A of transcripts showing increased TE is indeed independent of m6A erasers.
Western blots showing equal expression of FTO and Alkbh5 in GSCs and differentiated cells have been added to supplementary Figure 5B of the revised manuscript as the reviewer requested.

2. The authors present an interesting link between m6A and microRNA mediated recruitment of m6A erasers, FTO. It would be necessary to evaluate the protein levels of FTO in GSCs and following their differentiation.
Please see above.

3. The authors claim miRNAs mediated loss of m6A is dependent on their ability to recruit FTO to m6A bearing transcripts. It would be important to see if FTO KD could rescue the m6A loss and therefore reduce the increased TE in differentiated GSCs.
FTO KD rescues the increase in TE in differentiated GSCs. The data have been added to Figures 5E and 5F of the revised manuscript as the reviewer suggested.

Miscellaneous:

1. Figure 1B is not clear and legends seem invisible. It is unclear which genomic region is being looked at and therefore impossible to know if it is at all relevant to the differentiation of GSCs. It is unclear what is input and whether the data has been normalized. Scale of peaks in IGV is not shown so impossible to know even if the region is relevant (i.e. one of the transcripts bearing loss of m6A but whose translation is enhanced), whether the coverage plots are comparable.
The first diagram is not meant to show specific regions of interest but operates as a tool to aid the reader orient themselves. It is more of a methodological figure, summarizing project's direction. We have added scale of peaks and regions depicted as the reviewer suggested.

2. Figure 2A is unclear, a clear diagram would be presented.
3. Supp figure 1A is extremely difficult to read and understand.

We have modified the legends and Fonts of these Figures in the revised manuscript so they are clear to readers.

Please do not hesitate to contact me directly if you need additional information.

Sincerely,

Nikos Tapinos MD, PhD
