# Supplementary material for: miRNA-mediated loss of m6A increases nascent translation in glioblastoma
Source: PLoS Genet. 2021 Mar 8;17(3):e1009086. doi: 10.1371/journal.pgen.1009086 (PMC7971852; doi:10.1371/journal.pgen.1009086)
Supplement: Attachment — Submitted filename: Revision of plos genetic manuscript.docx [file pgen.1009086.s002.docx]

miRNA-mediated loss of m6A increases nascent translation in glioblastoma

Zepecki *et al* aimed to study the potential role of m6A modification during the differentiation of glioma stells cells in three glioblastoma patients. In this regard, Zepecki and colleagues compared the ribosomal profiles, transcriptome, and m6A methylome of GSCs and following their differentiation into three subtypes, mesenchymal, proneural and classical. Through comparative analysis, Zepecki *et al* report that m6A was noticeably reduced in transcripts that are most efficiently translated during differentiation. In order to understand whether this enhanced translation is relevant to the disease phenotype or a normal process of GSC differentiation, the authors should provide the same analysis in healthy control cells. In addition, the authors should consider providing western blot evaluation of m6A demethylases (ALKBH5/FTO) and see whether their conclusion that loss of m6A of transcripts showing increased TE is indeed independent of m6A erasers. Furthermore, the authors present an interesting link between m6A and microRNA mediated recruitment of m6A erasers, FTO. It would be necessary to evaluate the protein levels of FTO in GSCs and following their differentiation. The authors claim miRNAs mediated loss of m6A is dependent on their ability to recruit FTO to m6A bearing transcripts. It would be important to see if FTO KD could rescue the m6A loss and therefore reduce the increased TE in differentiated GSCs.

Miscellaneous

figure 1B is not unclear and legends seem invisible.

It is unclear which genomic region is being looked at and therefore impossible to know if it is at all relevant to the differentiation of GSCs. It is unclear what is input and whether the data has been normalized. In addition, scale of peaks in IGV is not shown so impossible to know even if the region is relevant (i.e. one of the transcripts bearing loss of m6A but whose translation is enhanced), whether the coverage plots are comparable.

Figure 2A is unclear, a clear diagram would be presented.

Supp figure 1A is extremely difficult to read and understand.
